# Testing the Ce Limit of Mass Bias Correction Using ^145^Nd/^142^Nd as Normalizing Ratio in Radiogenic Neodymium Isotope Analysis by MC‐ICP‐MS

**DOI:** 10.1002/rcm.9951

**Published:** 2024-12-06

**Authors:** Torben Struve, Martin Zander, Katharina Pahnke

**Affiliations:** ^1^ Institute for Chemistry and Biology of the Marine Environment (ICBM), School of Mathematics and Science Carl von Ossietzky Universität Oldenburg Oldenburg Germany

**Keywords:** isobaric interference, mass bias correction, MC‐ICP‐MS, neodymium isotopes

## Abstract

**Rationale:**

Neodymium isotopes are a powerful geochemical tool that has widely been used in terrestrial and extraterrestrial studies. Modern multicollector inductively coupled plasma mass spectrometers (MC‐ICP‐MS) allow fast, accurate, and precise analysis of the radiogenic Nd isotope ratio ^143^Nd/^144^Nd. These analyses comprise relatively high instrumental mass bias that is typically corrected for using the stable ^146^Nd/^144^Nd of 0.7219 and an exponential law. The instrument is usually tuned to optimize the operating conditions for isotope analysis, but this tuning is a trade‐off primarily between signal intensity, stability, and accuracy. Alternative, more effective approaches for mass bias correction have been proposed that use ^145^Nd/^142^Nd as normalizing ratio. However, one drawback of using this ratio is that the efficient removal of Ce from Nd is required to minimize the effect of isobaric interference of ^142^Ce on ^142^Nd.

**Methods:**

Here, we analyzed international Nd and rock reference materials using a Thermo Scientific Neptune *Plus* MC‐ICP‐MS to evaluate the sensitivity of ^145^Nd/^142^Nd‐based mass bias correction to varying Ce/Nd and in comparison with the commonly used ^146^Nd/^144^Nd‐based correction.

**Results:**

Our results show that the corrected ^143^Nd/^144^Nd of Ce‐doped JNdi‐1 and Ce‐containing USGS BCR‐2, NOD‐A‐1, and NOD‐P‐1 reference materials are insensitive to Ce/Nd of up to ~1.

**Conclusions:**

The correction of instrumental mass bias with ^145^Nd/^142^Nd as a normalizing ratio yields, as previously suggested, improved trueness and precision of ^143^Nd/^144^Nd data in comparison with ^146^Nd/^144^Nd‐based corrections, even under high Ce/Nd of ~1. This allows improved optimization of signal intensity during instrument tuning, which is particularly useful for natural samples with low Nd content. [Correction added on 10 December 2024, after first online publication: The Results and Conclusions in Abstract has been corrected in this version.]

## 1 Introduction

The variations of the radiogenic neodymium (Nd) isotope ratio ^143^Nd/^144^Nd are commonly investigated to study cosmological, geological, and environmental processes in natural samples. Although not trivial, the advent of multicollector inductively coupled mass spectrometry (MC‐ICP‐MS) allowed significantly faster analysis of the ^143^Nd/^144^Nd ratio than thermal ionization mass spectrometry (TIMS) established earlier. Early work on Nd isotope analysis with TIMS used different (nearly) stable Nd isotope ratios for correction of mass fractionation, including ^150^Nd/^142^Nd [1], ^146^Nd/^142^Nd [2], ^148^Nd/^144^Nd [3, 4], and ^146^Nd/^144^Nd [5]. Whereas the mass discrimination in TIMS is typically low, it is about one order of magnitude higher in MC‐ICP‐MS, which requires effective correction procedures. Different mathematical approaches have been suggested to correct for the instrumental mass bias in MC‐ICP‐MS, including the exponential law [6, 7], the power law [8, 9], and the general power law with empirical modification accounting for variable mass dependencies [10, 11]. Today, the routine analysis of the radiogenic ^143^Nd/^144^Nd ratio with both TIMS and MC‐ICP‐MS involves mass bias corrections using the exponential law and ^146^Nd/^144^Nd = 0.7219 as normalizing ratio [5]. Yet the instrumental mass bias in Nd isotope analysis with MC‐ICP‐MS is dependent on the plasma conditions [12, 13], which implies that MC‐ICP‐MS are “tuned” to optimize the (plasma) operating conditions, and in particular the gas flows, for high and stable signal intensity as well as accurate and precise results using ^146^Nd/^144^Nd = 0.7219 and the exponential law.

Over 30 years ago, Thirlwall [14] showed that the mass bias corrected Nd isotope data analyzed by TIMS can be improved by applying secondary corrections using ^142^Nd/^144^Nd. About a decade later, Vance and Thirlwall [15] suggested that a secondary empirical correction can also be applied to MC‐ICP‐MS Nd isotope data using the residual correlation in mass bias corrected ^142^Nd/^144^Nd and ^143^Nd/^144^Nd. This simple secondary mass bias correction improves significantly the trueness and precision of the final Nd isotope data [15]. The same authors also suggested that the mass bias correction via the exponential law is most successful when the average mass of the normalizing ratio is similar to the average mass of the normalized ratio. For example, the radiogenic ^143^Nd/^144^Nd ratio shares its average mass of 143.5 with ^145^Nd/^142^Nd. Yet these approaches are complicated by the fact that natural samples contain Ce, which has an isobaric interference with Nd on mass 142. So efficient wet‐chemical separation of Ce from Nd is critical for using any of these two approaches and, in particular, for a primary mass bias correction with ^145^Nd/^142^Nd as normalizing ratio. A (near) quantitative wet‐chemical removal of Ce from Nd has been achieved with methanol‐based high‐pressure liquid chromatographic (HPLC) techniques [16], α‐hydroxyisobutyric acid‐based approaches (e.g., [17, 18, 19]), and liquid–liquid microextraction (e.g., [20, 21]). However, these approaches involve poisonous reagents and/or require rigorous control of experimental conditions so that simple hydrochloric/nitric acid‐based separation techniques are often preferred for separating Nd from LREE. Although recent developments show promising results for separating Nd from sample matrix with TrisKem/Eichrom DGA resin (e.g., [22, 23]), Ln resin‐based approaches are commonly applied for wet‐chemical separation of Nd from sample matrix for radiogenic Nd isotope analysis [24, 25], typically in tandem with AG50W [26, 27, 28], or Eichrom/TrisKem TRU or RE resins [24, 25, 29]. However, a near‐quantitative separation of Nd from Ce using Ln resin is time‐consuming (e.g., [24, 29]) and usually unnecessary if Nd isotopes are measured as Nd^+^ in MC‐ICP‐MS, in which case the Nd separation from the LREE with Ln resin can be done in less than 5 h (e.g., [30]).

Here, we use Ce‐doped reference material JNdi‐1 to compare the performance of ^146^Nd/^144^Nd versus ^145^Nd/^142^Nd with an exponential law for the correction of instrumental mass bias under increasing Ce/Nd. Our results confirm previous suggestions that higher trueness and precision of mass bias corrected ^143^Nd/^144^Nd are obtained with ^145^Nd/^142^Nd as a normalizing ratio [15]. Notably, our work shows that the ^145^Nd/^142^Nd based mass bias correction can be applied safely up to Ce/Nd of ~1, that is, a range that can easily be achieved for a wide range of natural samples with fast Ln resin (TrisKem International) based and commonly used techniques for wet‐chemical separation of Nd from Ce.

## 2 Material and Methods

The JNdi‐1 reference material used for this study was prepared from Nd_2_CO_3_ (Split 1, Position 150) as provided by the Geological Survey of Japan [31]. All digestions and dilutions were prepared with HNO_3_ purified with the Savillex DST‐1000 system in the cleanroom facility at the Institute of Chemistry and Biology of the Marine Environment (ICBM), University of Oldenburg. A series of dilutions was prepared volumetrically containing ~5 and 10 ppb JNdi‐1 and varying amounts of commercially available Ce solution (CertiPUR®, Merck KGaA, Germany). USGS rock reference material BCR‐2 was digested following previously published procedures [32]. In brief, BCR‐2 rock powder was transferred into ultraclean PTFE vessels fitting the PicoTrace DAS 30 pressure digestion system. Sample digestion was achieved with a mixture of ultrapure concentrated HNO_3_ (Fisher Scientific OPTIMA^TM^), HF (Fisher Scientific OPTIMA^TM^), and HClO_4_ (Roth ROTIPURAN® Ultra). Ferromanganese crust powders NOD‐A‐1 and NOD‐P‐1 were leached with a mixture 0.005 M hydroxylamine hydrochloride, 1.5% acetic acid, and 0.001 M EDTA buffered to pH∼4 with suprapure ammonia [33]. The ion‐chromatographic separation of Nd from sample matrix for Nd isotope analysis followed previously published protocols using Biorad® AG1‐X8 resin and TrisKem Ln resin [30].

All Nd isotope analyses were carried out during two measurement sessions in December 2022 and in August 2024 using a Thermo Scientific Neptune *Plus* MC‐ICP‐MS at the ICBM equipped with Ni X‐skimmer and Jet‐sampler cones and nine Faraday cups connected to eight 10^11^ Ω amplifiers and one 10^12^ Ω amplifier connected to the cup for ^149^Sm. A gain calibration was carried out at the beginning of every session. The samples were introduced in 2% HNO_3_ matrix with a CETAC PFA nebulizer (nominal flow rate of 100 uL/min) into a CETAC Aridus II desolvating system, which was operated with sweep gas flows of ~5 L/min and N_2_ addition of 10 mL/min tuned to reduce fractionation effects that cannot be corrected with the commonly used mass fractionation laws [12]. In particular, “cold” plasma tends to allow higher oxide and hydride formation rates, thus increasing isobaric interferences that can be reduced by tuning the instrument to “hot” plasma conditions [13, 34]. The instrument's cool, auxiliary, and sample gas flows were set to 14.50, 1.00, and 1.03 L/min, respectively. Individual sample measurements comprised 48 cycles with an integration time of 4.194 s. Typical signal intensities were ~2–6 V on ^144^Nd, whereas background analytical blank signals were usually ~1 mV and thus not corrected for.

All data presented in this work were processed following the procedure used by the Thermo Scientific Neptune Method Editor software [35], thus allowing online monitoring of the results during the analysis. The correction of isobaric interferences from Ce and Sm isotopes included a mass bias factor calculated from the measured ^146^Nd/^144^Nd and the true ^146^Nd/^144^Nd of 0.7219. We used ^146^Nd/^144^Nd for Sm instead of ^149^Sm/^147^Sm to avoid artificial effects on mass 144 resulting from large inaccuracies of ^149^Sm/^147^Sm in samples that are virtually Sm‐free (<0.1 mV for ^147^Sm). Isobaric interferences of ^144^Sm on ^144^Nd and ^150^Sm on ^150^Nd were routinely monitored and corrected for by measuring ^147^Sm; monitoring of ^140^Ce allowed correcting the interference of ^142^Ce on ^142^Nd (see below).

During an initial step, the data processing routine approximates the measured ^146^Nd/^144^Nd by correcting for Sm without taking mass bias for Nd or Sm into account:

(1) $${\left(\frac{146_{\mathrm{Nd}}}{144_{\mathrm{Nd}}}\right)}_{\text{initial}}=\frac{{146_{\mathrm{Nd}}}_{\text{meas}.}}{\left({144_{\left(\mathrm{Nd}+\mathrm{Sm}\right)}}_{\text{meas}.}-\left(\frac{147_{{\mathrm{Sm}}_{\text{meas}.}}}{{\left(\frac{147_{\mathrm{Sm}}}{144_{\mathrm{Sm}}}\right)}_{\text{true}}}\right)\right)},$$

where ^147^Sm/^144^Sm_true_ = 4.83871. The subscripts “meas.” and “true” refer to the measured and abundance/consensus ratios, respectively. The initial ^146^Nd/^144^Nd is used to correct Sm for mass bias and calculate the “measured” ^147^Sm/^144^Sm:

(2) $${\left(\frac{147_{\mathrm{Sm}}}{144_{\mathrm{Sm}}}\right)}_{\text{meas}.}={\left(\frac{147_{\mathrm{Sm}}}{144_{\mathrm{Sm}}}\right)}_{\text{true}}\times {\left(\frac{{\left(\frac{146_{\mathrm{Nd}}}{144_{\mathrm{Nd}}}\right)}_{\text{initial}}}{{\left(\frac{146_{\mathrm{Nd}}}{144_{\mathrm{Nd}}}\right)}_{\text{true}}}\right)}^{\left(\frac{\ln\left(\frac{{\text{mass}}^{147_{\mathrm{Sm}}}}{{\text{mass}}^{144_{\mathrm{Sm}}}}\right)}{\ln\left(\frac{{\text{mass}}^{146_{\mathrm{Nd}}}}{{\text{mass}}^{144_{\mathrm{Nd}}}}\right)}\right)}.$$

The ^146^Nd/^144^Nd corrected for ^144^Sm interference is then calculated using ^149^Sm/^147^Sm_meas._ and Equation (1):

(3) $${\left(\frac{146_{\mathrm{Nd}}}{144_{\mathrm{Nd}}}\right)}_{\mathrm{int}.\text{corr}.}=\frac{{146_{\mathrm{Nd}}}_{\text{meas}.}}{\left({144_{\left(\mathrm{Nd}+\mathrm{Sm}\right)}}_{\text{meas}.}-\left(\frac{147_{{\mathrm{Sm}}_{\text{meas}.}}}{{\left(\frac{147_{\mathrm{Sm}}}{144_{\mathrm{Sm}}}\right)}_{\text{meas}.}}\right)\right)},$$

where the subscript “int.corr.” refers to interference corrected. The interference of ^150^Sm on ^150^Nd was corrected by calculating ^147^Sm/^150^Sm_meas_ according to Equation (2) using ^147^Sm/^150^Sm_true_ = 2.02703. It is noted that the signal intensity of < 0.1 mV for ^147^Sm implies an overall minor effect of ^144^Sm interference on ^144^Nd during our measurements. In the presence of Sm, the direct interference of ^144^Sm on ^144^Nd used to calculate the mass bias factor requires an iterative approach with Equations (2) and (3) or accurate measurement of ^149^Sm/^147^Sm to obtain interference‐free ^147^Sm/^144^Sm and ^146^Nd/^144^Nd (see, e.g., [35]).

The correction of ^142^Ce interference on ^142^Nd followed the same systematics as for Sm:

(4) $${\left(\frac{142_{\mathrm{Ce}}}{140_{\mathrm{Ce}}}\right)}_{\text{meas}.}={\left(\frac{142_{\mathrm{Ce}}}{140_{\mathrm{Ce}}}\right)}_{\text{true}}\times {\left(\frac{{\left(\frac{146_{\mathrm{Nd}}}{144_{\mathrm{Nd}}}\right)}_{\mathrm{int}.\text{corr}.}}{{\left(\frac{146_{\mathrm{Nd}}}{144_{\mathrm{Nd}}}\right)}_{\text{true}}}\right)}^{\left(\frac{\ln\left(\frac{{\text{mass}}^{142_{\mathrm{Ce}}}}{{\text{mass}}^{140_{\mathrm{Ce}}}}\right)}{\ln\left(\frac{{\text{mass}}^{146_{\mathrm{Nd}}}}{{\text{mass}}^{144_{\mathrm{Nd}}}}\right)}\right)},$$

where ^142^Ce/^140^Ce_true_ = 0.125768 (see also discussion below). Interference‐corrected ^142^Nd/^144^Nd and ^145^Nd/^142^Nd are then calculated by adapting Equation (3):

(5) $${\left(\frac{142_{\mathrm{Nd}}}{144_{\mathrm{Nd}}}\right)}_{\mathrm{int}.\text{corr}.}=\frac{{142_{\left(\mathrm{Nd}+\mathrm{Ce}\right)}}_{\text{meas}.}-\left(\frac{140_{{\mathrm{Ce}}_{\text{meas}.}}}{{\left(\frac{140_{\mathrm{Ce}}}{142_{\mathrm{Ce}}}\right)}_{\text{meas}.}}\right)}{{144_{\mathrm{Nd}}}_{\mathrm{int}.\text{corr}.}},$$

(6) $${\left(\frac{145_{\mathrm{Nd}}}{142_{\mathrm{Nd}}}\right)}_{\mathrm{int}.\text{corr}.}=\frac{{145_{\mathrm{Nd}}}_{\text{meas}.}}{\left({142_{\left(\mathrm{Nd}+\mathrm{Ce}\right)}}_{\text{meas}.}-\left(\frac{140_{{\mathrm{Ce}}_{\text{meas}.}}}{{\left(\frac{140_{\mathrm{Ce}}}{142_{\mathrm{Ce}}}\right)}_{\text{meas}.}}\right)\right)}.$$

We note that the Ce interference correction yields similar results with a mass bias factor calculated iteratively using measured ^145^Nd/^142^Nd and ^145^Nd/^142^Nd_true_ = 0.305125. This value is derived from the TIMS data reported by Vance and Thirlwall [15] and consistent with other more recent TIMS results (e.g., [29, 36]). Offline data processing showed that up to eight iterations are necessary until a constant mass bias value is reached when ^145^Nd/^142^Nd is used instead of ^146^Nd/^144^Nd to calculate a mass bias factor for ^142^Ce interference correction. Further details of the Ce interference correction are discussed below.

The interference corrected Nd isotope ratios are then corrected for mass bias using ^146^Nd/^144^Nd or ^145^Nd/^142^Nd and an exponential (kinetic) law:

(7a) $$f_{\mathrm{kin}\;\left(\frac{146}{144}\right)}=\frac{\ln\left(\frac{{\left(\frac{146_{\mathrm{Nd}}}{144_{\mathrm{Nd}}}\right)}_{\text{true}}}{{\left(\frac{146_{\mathrm{Nd}}}{144_{\mathrm{Nd}}}\right)}_{\mathrm{int}.\text{corr}.}}\right)}{\ln\left(\frac{{\text{mass}}^{146_{\mathrm{Nd}}}}{{\text{mass}}^{144_{\mathrm{Nd}}}}\right)},$$

(7b) $$f_{\mathrm{kin}\;\left(\frac{145}{142}\right)}=\frac{\ln\left(\frac{{\left(\frac{145_{\mathrm{Nd}}}{142_{\mathrm{Nd}}}\right)}_{\text{true}}}{{\left(\frac{145_{\mathrm{Nd}}}{142_{\mathrm{Nd}}}\right)}_{\mathrm{int}.\text{corr}.}}\right)}{\ln\left(\frac{{\text{mass}}^{145_{\mathrm{Nd}}}}{{\text{mass}}^{142_{\mathrm{Nd}}}}\right)},$$

(8) $${\left(\frac{1{\mathrm{xy}}_{\mathrm{Nd}}}{1{\mathrm{xy}}_{\mathrm{Nd}}}\right)}_{\mathrm{mbc}}={\left(\frac{1{\mathrm{xy}}_{\mathrm{Nd}}}{1{\mathrm{xy}}_{\mathrm{Nd}}}\right)}_{\mathrm{int}.\text{corr}.}\times {\left(\frac{{\text{mass}}^{1{\mathrm{xy}}_{\mathrm{Nd}}}}{{\text{mass}}^{1{\mathrm{xy}}_{\mathrm{Nd}}}}\right)}^{f_{\mathrm{kin}}},$$

where “mbc” refers to the final mass bias corrected Nd isotope ratios indicated by “1xy” with ^143^Nd/^144^Nd being the ratio of particular interest in this work. No further normalization or correction procedures were applied to the data.

## 3 Results and Discussion

**FIGURE 1:**
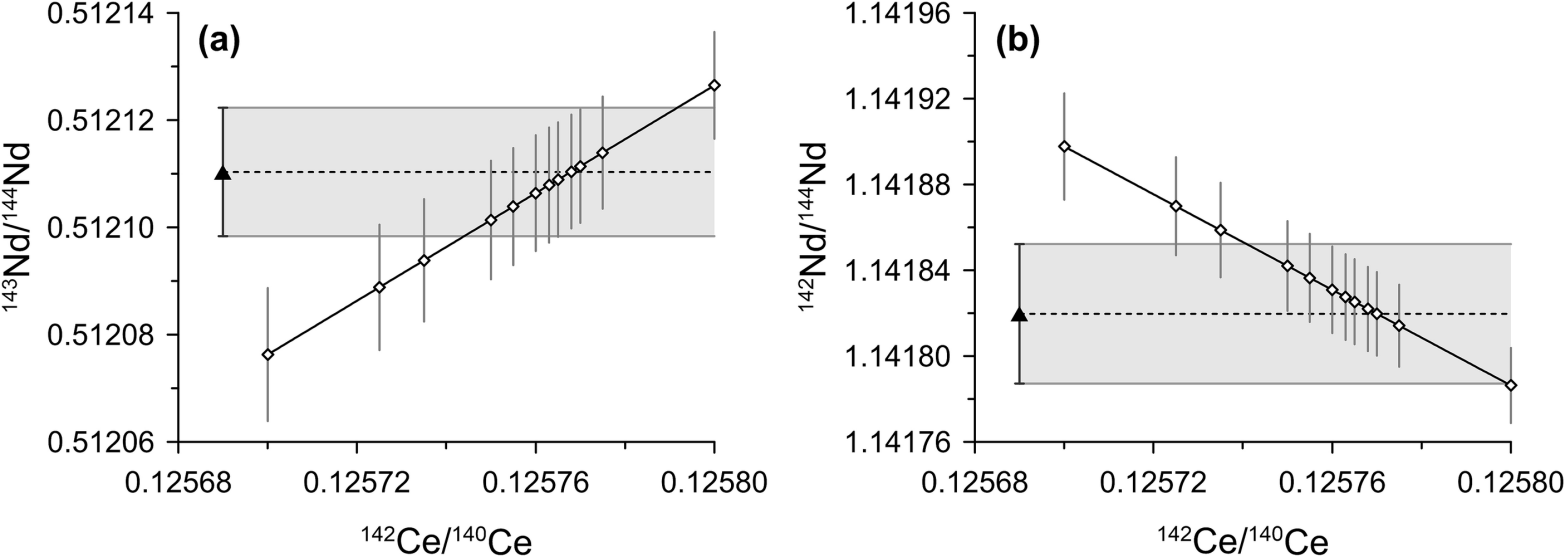
The influence of ^142^Ce/^140^Ce choice on mass bias corrected Nd isotope data using ^145^Nd/^142^Nd as a normalizing ratio. Results for (a) ^143^Nd/^144^Nd and (b) for ^142^Nd/^144^Nd. Nd isotope ratios and uncertainties are the averages and 2SD of repeated analysis (*n* = 4) of JNdi‐1 reference material at a concentration level of ~5 ppb Nd doped with 5 ppb Ce (^140^Ce/^144^Nd of 3.6). Average values of pure 5 ppb JNdi‐1 solution yielded (a) ^143^Nd/^144^Nd = 0.512110 ± 0.000012 and (b) ^142^Nd/^144^Nd = 1.141820 ± 0.000032 (*n* = 5, 2SD).

The effective correction of ^142^Ce isobaric interference on ^142^Nd is critical for using ^145^Nd/^142^Nd for mass bias correction of the radiogenic Nd isotope ratio of ^143^Nd/^144^Nd. Thirlwall and Anczkiewicz [37] reported ^142^Ce/^140^Ce varying from 0.125 for TIMS to 0.12584–0.12598 for the Micromass IsoProbe at Royal Holloway, University of London. These authors adjusted their ^142^Ce/^140^Ce used for interference correction so that the ^142^Nd/^144^Nd of Ce‐doped and Ce‐free Nd standard solutions match [37]. We adopted their approach and compared the ^145^Nd/^142^Nd‐normalized ^143^Nd/^144^Nd and ^142^Nd/^144^Nd of Ce‐doped JNdi‐1 with pure JNdi‐1 results. Analysis of Ce‐doped JNdi‐1 shows that both ^143^Nd/^144^Nd and ^142^Nd/^144^Nd are, as expected, sensitive to the choice of ^142^Ce/^140^Ce when ^145^Nd/^142^Nd is used as normalizing ratio (Figure 1a,b). Testing different ^142^Ce/^140^Ce for correcting the same raw data shows that ^142^Ce/^140^Ce = 0.125768 results in ^143^Nd/^144^Nd and ^142^Nd/^144^Nd for Ce‐doped JNdi‐1 that are indistinguishable from pure JNdi‐1 results measured in the same session (^143^Nd/^144^Nd = 0.512110 ± 0.000012, ^142^Nd/^144^Nd = 1.141820 ± 0.000032, 2SD of *n* = 5) (Figure 1). We therefore used this ratio for all Ce corrections carried out in this work and note that this ratio should be determined individually for other mass spectrometers. The ^142^Ce/^140^Ce may also be refined on a session‐by‐session basis to account for varying instrument operating conditions.

**FIGURE 2:**
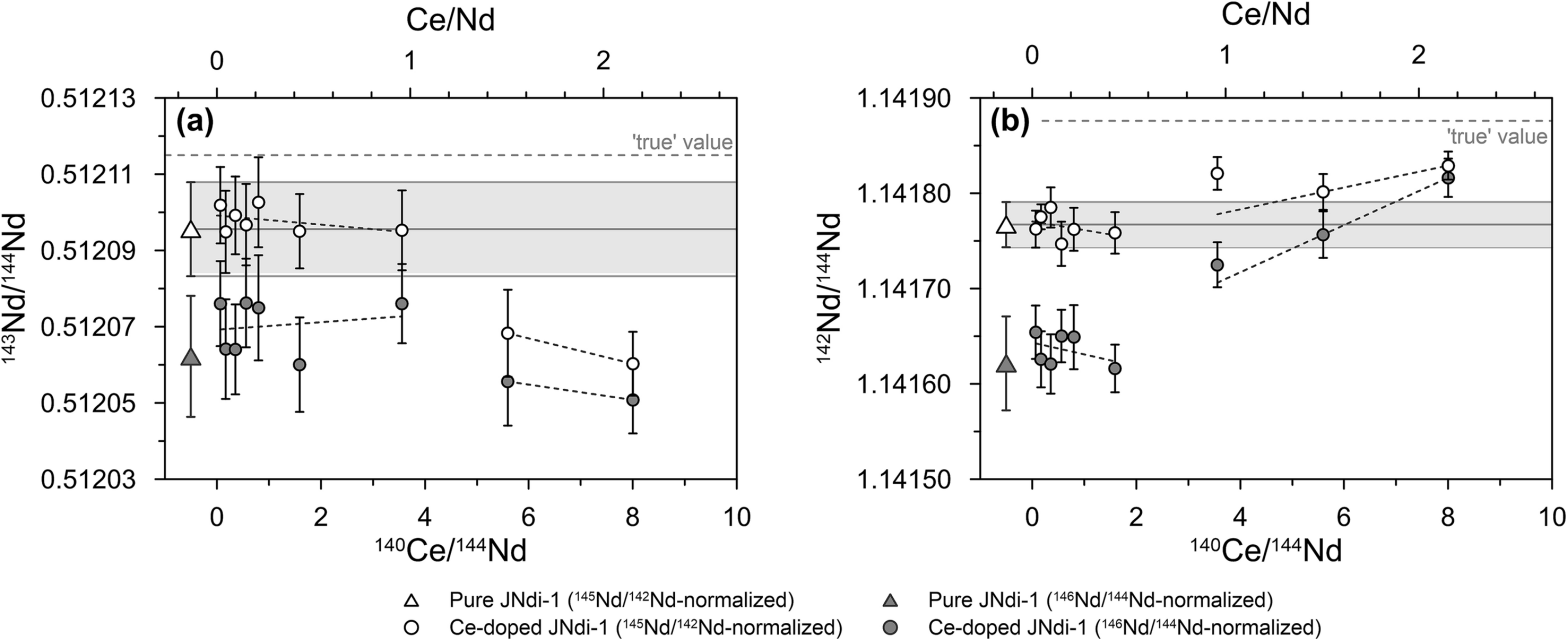
The limit of Ce for ^145^Nd/^142^Nd‐based mass bias correction using optimized ^142^Ce/^140^Ce. Dilution series including pure (*n* = 16, averages shown as triangles and stippled lines; gray bars represent the 2SD) JNdi‐1 bracketing Ce‐doped (circles) 10 ppb Nd JNdi‐1 solutions. (a) ^143^Nd/^144^Nd results for mass bias correction using ^145^Nd/^142^Nd = 0.305125 (white‐filled circles) and ^146^Nd/^144^Nd = 0.7219 (gray‐filled circles) as normalizing ratios. (b) As in (a), but for ^142^Nd/^144^Nd. Error bars of individual measurements (circles) represent the 2SE internal error (calculated from 48 cycles à 4.194 s). The “true” values are ^143^Nd/^144^Nd = 0.512115 [31] and ^142^Nd/^144^Nd = 1.141876 [14] in panels (a) and (b), respectively. Note that all data result from a single mass bias correction without any secondary corrections.

**FIGURE 3:**
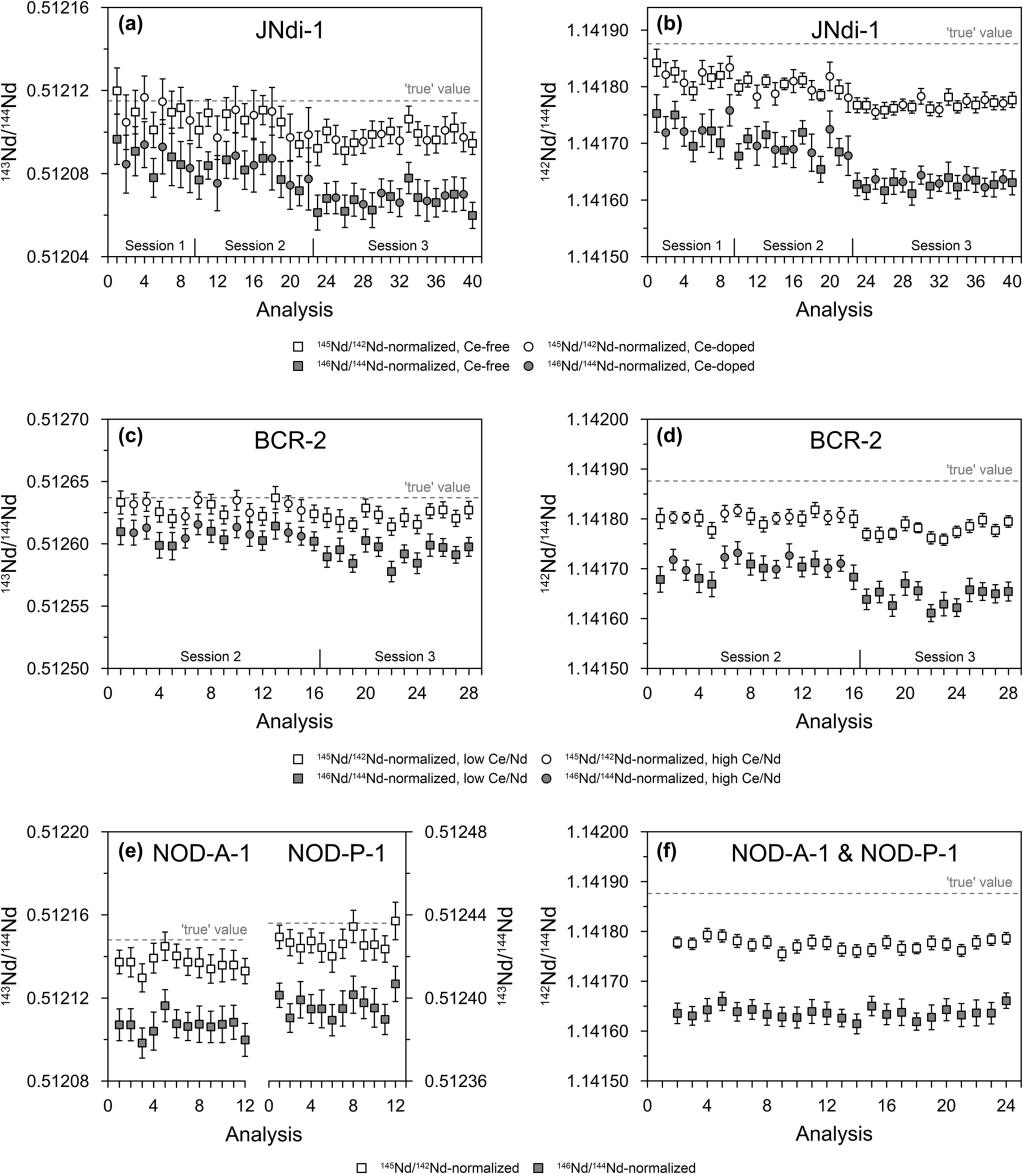
Results for single ^146^Nd/^144^Nd‐ and ^145^Nd/^142^Nd‐based mass bias correction using optimized ^142^Ce/^140^Ce. Results of Ce‐doped and pure JNdi‐1 solutions for (a) ^143^Nd/^144^Nd and (b) ^142^Nd/^144^Nd analyzed in three individual sessions with different Nd concentrations (5–20 pbb) and Ce/Nd between 0 and ~1. BCR‐2 results for (c) ^143^Nd/^144^Nd and (d) ^142^Nd/^144^Nd analyzed in Sessions 2 and 3. (e) ^143^Nd/^144^Nd and (f) ^142^Nd/^144^Nd results for NOD‐A‐1 and NOD‐P‐1 analyzed in Session 3. The stippled lines indicate “true” values: (a) ^143^Nd/^144^Nd = 0.512115 for JNdi‐1 [31], (c) ^143^Nd/^144^Nd = 0.512637 for BCR‐2 [38], and (e) ^143^Nd/^144^Nd = 0.512148 and ^143^Nd/^144^Nd = 0.512436 for NOD‐A‐1 and NOD‐P‐1, respectively [39]. Stippled lines in (b), (d), and (f) represent “true” ^142^Nd/^144^Nd of 1.141876 [14]. See Table 1 and the Supporting Information for more details. Note that all data result from a single mass bias correction without any secondary corrections. Error bars represent the 2SE internal error of the individual measurements (calculated from 48 cycles à 4.194 s).

Using ^142^Ce/^140^Ce = 0.125768, the ^143^Nd/^144^Nd results of our series of Ce‐doped JNdi‐1 solutions indicate that an effective correction of mass‐dependent fractionation is achieved with ^145^Nd/^142^Nd as a normalizing ratio for Ce/Nd up to ~1 (^140^Ce/^144^Nd of 3.6), whereas JNdi‐1 with Ce/Nd higher than ~1 show a shift to lower values (Figure 2a). We find elevated ^142^Nd/^144^Nd for ^140^Ce/^144^Nd of 3.6, which is slightly outside the analytical uncertainty when considering the 2SD for Ce‐free JNdi‐1 (see Figure 2b and the Supporting Information). Yet a possible influence of high Ce/Nd and insufficient correction of the ^142^Ce interference on ^142^Nd shows no measurable effect on ^143^Nd/^144^Nd for Ce/Nd of ~1 (Figure 2a). Therefore, we consider the ^145^Nd/^142^Nd‐based mass bias correction effective for Ce/Nd of up to ~1 (^140^Ce/^144^Nd of 3.6). This conclusion is further supported by ^145^Nd/^142^Nd‐normalized ^143^Nd/^144^Nd and ^142^Nd/^144^Nd results showing no difference for pure and Ce‐doped JNdi‐1 with Ce/Nd of 0.9 (Session 1) and 0.4 (Sessions 2 and 3) (Table 1, Figure 3a,b, and the Supporting Information). The similar ^143^Nd/^144^Nd and ^142^Nd/^144^Nd for USGS rock reference material BCR‐2 with high and low Ce/Nd (Figure 3c,d) as well as the results for NOD‐A‐1 and NOD‐P‐1 (Figure 3e,f) demonstrate that this correction can also be applied successfully to natural samples with variable Ce/Nd after ion‐exchange chromatography.

**TABLE 1:** Neodymium isotope results for JNdi‐1 and rock reference materials BCR‐2, NOD‐A‐1, and NOD‐P‐1 containing different amounts of Ce.

	n	Normalizing ratio ^146^Nd/^144^Nd							Normalizing ratio ^145^Nd/^142^Nd					
		^140^Ce/^144^Nd	^142^Nd/^144^Nd	2SD	^143^Nd/^144^Nd	2SD	^150^Nd/^144^Nd	2SD	^142^Nd/^144^Nd	2SD	^143^Nd/^144^Nd	2SD	^150^Nd/^144^Nd	2SD
**JNdi‐1 ref. values** ^a^		0.0	1.141876	0.000009^b^	0.512115	0.000007	0.236446	0.000006^b^	1.141876	0.000009^b^	0.512115	0.000007	0.236446	0.000006^b^
JNdi‐1 Sessions 1 & 2														
JNdi‐1	12	0.0	1.141706	0.000055	0.512084	0.000013	0.236362	0.000021	1.141810	0.000031	0.512107	0.000012	0.236299	0.000032
JNdi‐1 + Ce	6	1.6	1.141693	0.000030	0.512081	0.000011	0.236355	0.000016	1.141795	0.000028	0.512104	0.000012	0.236295	0.000016
JNdi‐1 + high Ce	4	3.3	1.141730	0.000033	0.512088	0.000010	0.236370	0.000015	1.141822	0.000020	0.512110	0.000011	0.236317	0.000016
JNdi‐1 Session 3														
JNdi‐1	11	0.0	1.141626	0.000016	0.512067	0.000010	0.236334	0.000009	1.141767	0.000013	0.512098	0.000009	0.236249	0.000007
JNdi‐1 + Ce	7	1.6	1.141634	0.000013	0.512068	0.000004	0.236334	0.000004	1.141770	0.000018	0.512097	0.000004	0.236252	0.000010
JNdi‐1 recalculated														
JNdi‐1 (23.11.2012)	14	0.0	1.141630	0.000056	0.512071	0.000018	0.236320	0.000020	1.141785	0.000020	0.512104	0.000013	0.236230	0.000029
JNdi‐1 (28.11.2012)	11	0.0	1.141651	0.000046	0.512066	0.000020	0.236358	0.000014	1.141754	0.000039	0.512089	0.000019	0.236296	0.000019
**BCR‐2 ref. values** ^**a**^			1.141876	0.000009^b^	0.512637	0.000012	0.236446	0.000006^b^	1.141876	0.000009^b^	0.512637	0.000012	0.236446	0.000006^b^
BCR‐2 (low Ce, session 2)	8	0.1	1.141694	0.000031	0.512605	0.000011	0.236354	0.000011	1.141800	0.000021	0.512628	0.000011	0.236292	0.000011
BCR‐2 (Ce, session 2)	8	1.7	1.141713	0.000025	0.512610	0.000007	0.236356	0.000010	1.141806	0.000010	0.512630	0.000009	0.236298	0.000013
BCR‐2 (low Ce, session 3)	12	< 0.1	1.141643	0.000034	0.512592	0.000014	0.236333	0.000011	1.141777	0.000024	0.512621	0.000010	0.236252	0.000011
**NOD‐A‐1 ref. values** ^**a**^			1.141876	0.000009^b^	0.512148	0.000008	0.236446	0.000006^b^	1.141876	0.000009^b^	0.512148	0.000008	0.236446	0.000006^b^
NOD‐A‐1 (session 3)		< 0.2	1.141638	0.000017	0.512106	0.000009	0.236334	0.000008	1.141777	0.000018	0.512137	0.000007	0.236251	0.000013
**NOD‐P‐1 ref. values** ^**a**^			1.141876	0.000009^b^	0.512436	0.000008	0.236446	0.000006^b^	1.141876	0.000009^b^	0.512436	0.000008	0.236446	0.000006^b^
NOD‐P‐1 (session 3)		< 0.1	1.141635	0.000025	0.512396	0.000010	0.236333	0.000009	1.141771	0.000017	0.512427	0.000009	0.236250	0.000012

^a^ ^143^Nd/^144^Nd reference values for JNdi‐1, BCR‐2, NOD‐A‐1, and NOD‐P‐1 from Tanaka et al. [31], Weis et al. [38], and Foster and Vance [39], respectively. Other ratios from Thirlwall [14].

^b^ Reported as 2SE uncertainty for recalculated values [2, 14].

Furthermore, our results confirm the findings of Vance and Thirlwall [15] that a similar average mass of normalizing and normalized ratios yields higher trueness and precision of the normalized ratio. That is, for lower mass Nd ratios of interest including ^143^Nd/^144^Nd, using a normalizing ratio of ^145^Nd/^142^Nd tends to yield higher trueness and precision than the normalization to ^146^Nd/^144^Nd = 0.7219 (Figures 2 and 3). In our study, the average ^143^Nd/^144^Nd of pure and Ce‐doped JNdi‐1 normalized to ^145^Nd/^142^Nd is 0.512103 ± 0.000014 (2SD, *n* = 40; for the three sessions shown in Figure 3a). Within analytical uncertainty, this mass bias corrected value is in agreement with the reference value of Tanaka et al. [31] (^143^Nd/^144^Nd = 0.512115 ± 0.000007). Interestingly, our value is even closer to the slightly lower preliminary ^143^Nd/^144^Nd of 0.512104 [40] and the long‐term TIMS average of ^143^Nd/^144^Nd = 0.512107 ± 0.000028 used as JNdi‐1 benchmark value by Yu et al. [13]. Normalizing the same data to ^146^Nd/^144^Nd = 0.7219 yields lower ^143^Nd/^144^Nd of 0.512076 ± 0.000020 (Figure 3a). In addition, the 2SE of individual sample measurements show only slightly but systematically lower 2SE when using ^145^Nd/^142^Nd (average 2SE of 0.000008 for *n* = 40 JNdi‐1 measurements) as normalizing ratios as compared to using ^146^Nd/^144^Nd (average 2SE of 0.000010). The difference is small, but results from the two different correction procedures applied to the exact same raw data. Lower 2SE are expected from a more effective mass bias correction with ^145^Nd/^142^Nd as normalizing ratio applied on a cycle‐by‐cycle basis. Due to the higher mass difference, the differences in trueness and precision between the two normalizing ratios are more pronounced in the mass bias corrected ^142^Nd/^144^Nd results. The average ^142^Nd/^144^Nd of 1.141790 ± 0.000048 has lower 2SD (*n* = 40) and is closer to the ‘true’ TIMS value of 1.141876 [2, 14, 15] than the ^142^Nd/^144^Nd of 1.141672 ± 0.000087 using ^146^Nd/^144^Nd as normalizing ratio (Figure 3b). Similarly, the mass bias correction with ^145^Nd/^142^Nd as normalizing ratio yields systematically lower 2SE for individual ^142^Nd/^144^Nd measurements (average 2SE of 0.000016, *n* = 40) than ^146^Nd/^144^Nd‐based correction (average 2SE of 0.000024, *n* = 40). Only for the higher Nd mass ratios such as ^150^Nd/^144^Nd, ^146^Nd/^144^Nd is more effective as a normalizing ratio (Table 1 and the Supporting Information).

It is noted that the observed differences between the two mass bias correction approaches could generally also be related to Faraday cup deterioration [14, 41] and/or fractionation processes unaccounted for by standard mass bias correction approaches [12, 42]. Using the same procedure outlined in Section 2, reanalysis of Nd isotope data measured at the ICBM in November 2012 soon after instrument installation and published later by Basak et al. [43, 44] reproduce the results presented in this study (Table 1 and the Supporting Information), thus suggesting that Faraday cup deterioration is unable to explain the systematic differences between the two normalizing approaches. The analysis in 2012 used an H‐skimmer and Jet‐sampler cone setup. Considering that unaccounted fractionation processes in Nd isotope analysis are probably largely related to NdO^+^ formation at the interface (and thus sensitive to the choice of cones) [12], the reproducibility of improved Nd mass bias correction using ^145^Nd/^142^Nd as normalizing ratio is not an artifact of instrument setup and/or plasma operating conditions when using a CETAC Aridus II desolvating system. Moreover, the formation of NdO^+^ is expected to be influenced by sample matrix [12], but our results for sample solutions with different matrices (high and low Ce BCR‐2, NOD‐A‐1, NOD‐P‐1, pure, and Ce‐doped JNdi‐1) show similar systematics for the two mass bias correction approaches further supporting that such mass‐independent fractionation processes are unable to explain the systematic improvement using ^145^Nd/^142^Nd as compared to ^146^Nd/^144^Nd for Nd mass bias correction. Accordingly, the uncorrected ^145^Nd/^142^Nd and ^146^Nd/^144^Nd show no indication for significant mass‐independent fractionation (correlation coefficient *R* ^2^ of 0.99949 for *n* = 92 JNdi‐1, NOD‐A‐1, NOD‐P‐1, and BCR‐2 analyses listed in Table 1 and in the Supporting Information).

Importantly, the maximum Ce/Nd we tested is significantly higher than Ce/Nd of < 0.5 typically achieved with our separation procedure using Ln resin (TrisKem International) for different environmental samples including marine sediments, rock powders, and (ferro)manganese precipitates (Table 1; [30]) that often show naturally elevated Ce/Nd as reflected by positive Ce anomalies [45, 46]. Yet testing different Ce/Nd before using ^145^Nd/^142^Nd routinely as normalizing ratio and including Ce‐doped JNdi‐1 measurements is essential for Nd isotope runs using correction involving ^142^Nd. The Ce/Nd of monitoring solutions may be adjusted depending on the typical wet‐chemical Ce‐Nd separation efficiency. It has been recognized that higher signal intensities in isotope analysis with MC‐ICP‐MS are often related to higher instrumental mass bias with instrument tuning typically targeting the best compromise of signal intensity, stability, and reproducibility of reference values (e.g., [12]). Therefore, the better performance of ^145^Nd/^142^Nd as a normalizing ratio for mass bias correction implies that reference values can be reproduced (and samples measured) at overall higher signal intensities. That is, the ^145^Nd/^142^Nd‐based mass bias correction is particularly advantageous for the analysis of samples with low Nd content due to the better trueness and precision paired with a tolerance of high sample Ce/Nd allowing overlapping Ce and Nd elution peaks, and thus optimizing Nd recovery and time consumption for Ln resin‐based Nd separation (cf. [28, 29, 32]). In comparison to recently published work proposing a regression correction of instrumental mass bias [13], the ^145^Nd/^142^Nd‐based approach allows tracking the isotope ratios online during tuning and measurement with minimal offline post‐processing of the data. We note that systematic offsets and analytical drift can occur in the mass bias corrected ^143^Nd/^144^Nd with either correction approach and may be corrected for using secondary normalization procedures (e.g., [14, 15, 37]).

Importantly, two potential caveats need to be considered when using the ^142^Nd‐based mass bias correction approaches. Deviations from stable terrestrial ^142^Nd/^144^Nd related to nucleosynthetic isotope variations and/or the decay of the short‐lived isotope ^146^Sm (T_½_ ~103 Ma) after silicate Sm/Nd differentiation of the Early Earth may compromise the trueness of ^142^Nd‐based mass bias correction. Yet the small variations (< 15–20 ppm for ^142^Nd/^144^Nd; [20, 36, 47, 48, 49]) are typically similar to analytical uncertainty for ^142^Nd/^144^Nd (see the Supporting Information and Table 1) and are relevant only in very specific terrestrial and nonterrestrial sample material (e.g., [20, 36, 47, 48, 49]). Another potential caveat is Ce isotope fractionation in natural samples and/or during ion‐chromatographic separation. Ferromanganese crusts like NOD‐A‐1 and NOD‐P‐1 are among the most fractionated natural sample materials reflected by δ^142^Ce of ~0.12‰ and ~0.14‰, respectively [50]. However, our ^143^Nd/^144^Nd and ^142^Nd/^144^Nd results for NOD‐A‐1 and NOD‐P‐1 show no indication for a significant effect of natural Ce isotope variations on ^145^Nd/^142^Nd mass bias correction (see Figure 3e,f). Notably, Ce elution from Ln resin causes Ce isotope fractionation [51]. Incomplete removal of Ce from Nd is a characteristic of our Ln‐based Nd separation protocol, so we expect that Ce isotope fractionation is largest in the samples with the lowest Ce/Nd. That is, for our BCR‐2 with Ce/Nd of ~0.03 (NOD‐A‐1 and NOD‐P‐1 accordingly; see the Supporting Information), we calculate Ce removal of ~98%, based on published Ce/Nd concentrations of BCR‐2 [52] and Ce/Nd in our BCR‐2 fractions analyzed for their Nd isotope compositions. This degree of Ce removal would imply δ^142^Ce on the order of ~ −0.85‰ [51], far exceeding natural variations [50]. However, the results for different BCR‐2 aliquots with different Ce/Nd (and hence different Ce isotope fractionation) show remarkably reproducible ^142^Nd/^144^Nd and ^143^Nd/^144^Nd when using ^145^Nd/^142^Nd as a normalization ratio during mass bias correction (Figure 3c,d). As such, our results demonstrate that natural and laboratory‐induced Ce isotope fractionation has no significant effect on the final, corrected Nd isotope data, thus providing also guidance for elevated Ce/Nd permitting the application of secondary corrections using ^142^Nd/^144^Nd (e.g., [14, 15, 37]).

## 4 Conclusions

We show that optimization of ^142^Ce/^140^Ce allows for the correction of isobaric interference of ^142^Ce on ^142^Nd for to Ce/Nd levels of up to ~1 when using ^145^Nd/^142^Nd as normalizing ratio for instrumental mass bias correction of ^143^Nd/^144^Nd analyzed with MC‐ICP‐MS. Our data confirm previous work that suggested that ^145^Nd/^142^Nd‐based mass bias corrections produce results with higher trueness and precision than the more commonly applied ^146^Nd/^144^Nd ratio. Therefore, this approach is considered a powerful alternative and/or addition to the ^146^Nd/^144^Nd‐based mass bias correction during Nd isotope analysis. It is easy to implement and the tolerance for high Ce/Nd is particularly useful for samples with low Nd content allowing overlapping Ce and Nd elution peaks during the wet‐chemical separation of the two elements and thus improving the recovery of Nd. Moreover, the better performance of mass bias correction using ^145^Nd/^142^Nd as normalizing ratio also provides the advantage of improving uncertainties in low concentration Nd isotope analysis and it requires less tuning to optimize instrument operating conditions for Nd isotope analysis.

## Author Contributions

**Torben Struve:** conceptualization, formal analysis, writing – original draft, visualization, writing – review and editing, investigation, methodology, validation, data curation. **Martin Zander:** conceptualization, data curation, writing – review and editing, investigation. **Katharina Pahnke:** conceptualization, writing – review and editing, funding acquisition, investigation, resources.

## Conflicts of Interest

The authors declare no conflicts of interest.

## Supporting information


**Data S1.** Supporting Information

## Data Availability

The data that support the findings of this study are available in the Supporting Information of this article.
